# Simulated and clinical aerosol spread in common periodontal aerosol-generating procedures

**DOI:** 10.1007/s00784-022-04532-8

**Published:** 2022-05-17

**Authors:** Anthony Puljich, Kexin Jiao, Ryan S. B. Lee, Laurence J. Walsh, Sašo Ivanovski, Pingping Han

**Affiliations:** The University of Queensland, School of Dentistry, Brisbane, QLD 4006 Australia

**Keywords:** Air polisher, Splatter spread, Aerosol particles, Dental AGPs, Clinical bioaerosol spread

## Abstract

**Objectives:**

This study evaluated particle spread associated with various common periodontal aerosol-generating procedures (AGPs) in simulated and clinical settings.

**Materials and methods:**

A simulation study visualized the aerosols, droplets, and splatter spread with and without high-volume suction (HVS, 325 L/min) during common dental AGPs, namely ultrasonic scaling, air flow prophylaxis, and implant drilling after fluorescein dye was added to the water irrigant as a tracer. Each procedure was repeated 10 times. A complementary clinical study measured the spread of contaminated particles within the dental operatory and quantified airborne protein dispersion following 10 min of ultrasonic supragingival scaling of 19 participants during routine periodontal treatment.

**Results:**

The simulation study data showed that air flow produced the highest amount of splatters and the ultrasonic scaler generated the most aerosol and droplet particles at 1.2 m away from the source. The use of HVS effectively reduced 37.5–96% of splatter generation for all three dental AGPs, as well as 82–93% of aerosol and droplet particles at 1.2 m for the ultrasonic scaler and air polisher. In the clinical study, higher protein levels above background levels following ultrasonic supragingival scaling were detected in fewer than 20% of patients, indicating minimal particle spread.

**Conclusions:**

While three common periodontal AGPs produce aerosols and droplet particles up to at least 1.2 m from the source, the use of HVS is of significant benefit. Routine ultrasonic supragingival scaling produced few detectable traces of salivary protein at various sites throughout the 10-min dental operatory.

**Clinical relevance:**

The likelihood of aerosol spread to distant sites during common periodontal AGPs is greatly reduced by high-volume suction. Clinically, limited evidence of protein contaminants was found following routine ultrasonic scaling, suggesting that the the majority of the contamination consisits of the irrigant rather than organic matter from the oral cavity.

**Supplementary Information:**

The online version contains supplementary material available at 10.1007/s00784-022-04532-8.

## Introduction

The current SARS-CoV-2 global pandemic has renewed interest in the spread of splatter, droplets, and aerosols in dental clinical settings [1, 2]. Since particles, particularly aerosols, can transmit this disease to people, controlling the spread of aerosolized particles has become one of the core strategies for reducing occupationally acquired infections with the SARS-CoV-2 virus [3]. The World Health Organization (WHO) defines splatter as particles greater than 100 μm in size, droplets as particles between 5 and 100 μm in size, and aerosols as particles smaller than 5 μm (https://www.who.int/news-room/commentaries/detail/transmission-of-sars-cov-2-implications-for-infection-prevention-precautions). Splatter, droplets, and aerosols can be produced during normal physiological activities, such as breathing, speaking, coughing, and sneezing [4], as well as by aerosol-generating procedures (AGPs) performed as part of dental treatment (e.g. root debridement using ultrasonic scalers) [5].

Dental procedures generate particles that are a mixture of saliva, blood, water coolant, plaque, gingival crevicular fluid, tooth hard tissue debris, calculus, and dental restorative materials that generate potential hazards to dental professionals [6, 7]. The extent and spread pattern of common dental AGPs need to be identified before applying mitigating strategies. A recent systematic review included 48 studies of dental AGPs and it suggests that ultrasonic scaling (44 studies) and high-speed handpiece (31 studies) are the most common dental AGPs that have been investigated particles spread, with less attention in low-speed implant drills (4 studies) and air polisher (4 studies) [8]. Among common periodontal AGPs, a low-speed implant drill with water irrigation is often used for dental implant osteotomy preparation during dental implant surgery [9]; however, limited evidence exists for visualizing in vitro particles travel under a low-speed implant drill, which is considered an aerosol producing procedure and was hence subject to public health order restrictions during the COVID-19 pandemic. Moreover, we and other researchers showed that ultrasonic scalers generate the largest amount of particle spread compared to high-speed and low-speed handpieces in simulated laboratory settings, either using bacteria colony-forming units (CFUs) on an agar plate [10, 11] or fluorescence tracing dye [4, 12, 13]. Limited studies determined the visualized particle pattern with and without high-volume suction during common but diverse dental AGPs, namely ultrasonic scaler, low-speed implant drill, and air polisher, which were performed in this study. Meanwhile, some prevention measures are proven to significantly reduce splatter or aerosol spread [13–18], such as medium-volume suction (159 L/min in [13]), unstated speed of HVS [16], and mechanical extraction [15]. It is essential to investigate the efficacy of high-volume suction (> 250 L/min defined by the BS EN ISO 10637 standard) on splatter, droplets, and aerosols spread in an in vitro setting, an aspect that is overlooked in most recent studies.

Most of the current studies investigate particle spread and aerosol mitigation strategies for in vitro studies either in a physical containment level 2 (PC2) laboratory or simulated clinical settings (reviewed in [8]). An in vitro study showed that ultrasonication with mouthwash—hydrogen peroxide—can reduce aerosols biofilm CFU following the removal of dental biofilms [19]. A recent systematic review consisting of 17 clinical studies with 724 patients [18] concluded that the use of HVS and pre-procedure mouth rinse can reduce bacterial CFU formation in bioaerosols generated by high-speed rotary instruments. A clinical study by Meethil et al. used high-speed handpieces and ultrasonic scaling with high-volume intra-oral evacuators (7.1 L/min). The trace of aerosol bacteria by 16S sequencing was found to mainly originate from the dental irrigant, not from saliva [10] suggesting that dental treatment might not be a factor in increasing the risk for infectious virus transmission. However, since dental aerosols contain more than microorganisms, a case can be made to utilize an alternative relevant method (i.e., protein contamination) to detect bioaerosol distribution under routine treatment when both HVS and pre-procedure mouth rinse are applied.

Here, we aim to expand existing knowledge on visualizing the distribution of particles following common dental AGPs in an in vitro setting with and without HVS and determine the particles spread during non-surgical periodontal treatment for 19 patients using an ultrasonic scaler in a clinical setting.

## Materials and methods

This study explored the generation and spread of particles created by dental AGPs in both simulated laboratory and clinical environments. The in vitro simulated experiment investigated the splatter, droplet, and aerosol spread patterns in a physical containment level 2 (PC2) laboratory setting with three common dental AGPs (air polishing, ultrasonic scaling, and implant drilling) with and without HVS. In the in vitro simulated part, distilled water containing fluorescein was used for all three periodontal AGPs in the water coolant. The clinical study explored bioaerosol travel for patients undergoing non-surgical periodontal treatment using supragingival ultrasonic scaling.

### Simulated splatter, droplets, and aerosol with HVS

#### Simulation study experimental setup

The experiment protocol was based on that reported recently by our group [5] and is described in Supplementary Figure 1. The experiment was carried out in a 25 m^2^ room which had 7 air changes per hour and was located within a PC2 laboratory at the UQ’s School of Dentistry. A Columbia phantom head mannequin (One Dental, Castle Hill, NSW, Australia) containing typodont teeth in both jaws was used. Mock dental procedures were performed on the mandibular right central incisor (tooth 41, FDI World Dental Federation notation). Fluorescein sodium salt (Sigma-Aldrich, St Louis, MO, USA) was added to the water coolant reservoirs of dental devices at a final concentration of 1 mg/mL (approximately 3.0 mMol/L) as a tracer dye to track particle travel. To prevent bias, one periodontist trainee (A.P.) performed all the simulated and clinical experiments.

The following dental devices were used for the mock periodontal AGPs:

- An ultrasonic piezoelectric scaler (EMS Piezon, Nyon, Switzerland) was used at intensity setting 10 and water flow rate at 48 mL/min, with a scaler tip of type PS. For the experimental protocol, the scaler tip was positioned adjacent to the lingual surface of tooth 41.
- An air polisher device (EMS Air Flow Prophylaxis Master, Nyon, Switzerland), was used at an air pressure setting of 3 (1.9 Bar), with a water flow setting of 70% at 53 mL/min. The abrasive particles were 14-μm erythritol powder. The tip of the air-polishing device was located 3–5 mm from the buccal aspect of tooth 41, at an angle of approximately 45 degrees, with the spray aimed towards the incisal edge.
- A 2.2-mm diameter dental implant osteotomy drill (Straumann Holding AG, Basel, Switzerland) was used in a 20:1 reduction handpiece (W&H Dentalwerk, Burmoos, Austria) at 200 revolutions per minute. The water coolant flow rate was 100 mL/min. For this experiment, tooth 41 was removed from the typodont model and the implant drill was placed at 2 mm along the imaginary line joining the incisal edges of the 31 and 42 teeth.

Each device was tested without suction to establish baseline data and then once again using intra-oral high-volume evacuation (Aspi-Jet 6, Cattani S.p.A, Parma, Italy) with an airflow of approximately 325 L/min (measured with an airflow device, Durr Dental, Bietigheim-Bissingen, Germany). The suction used was comparable to HVS used in the clinical setting. The evacuation tip was held approximately 10 mm from tooth 41, favoring the left side of the mannequin. For the ultrasonic handpiece, the procedure was carried out for 10 min, while the air-polishing and implant surgical drill procedures lasted 2 min each to mimic a real clinical scenario. For the air-polishing device, the suction tip was placed adjacent to the point on the tooth where the powder made contact. Each AGP was repeated 10 times for each scenario (without or with HVS).

**Fig. 1:**
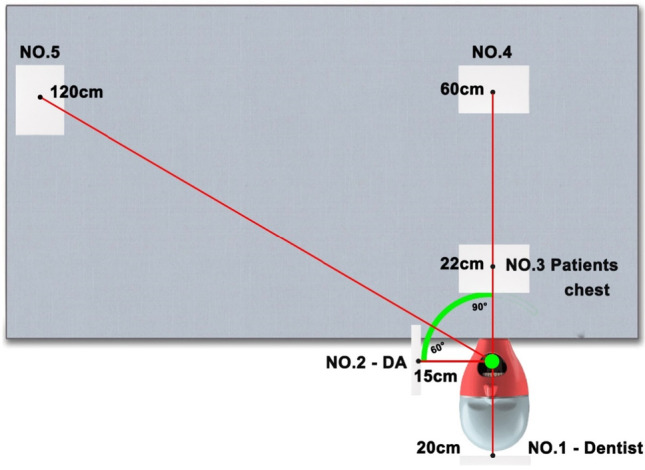
The five locations of the filter paper strips were used to collect in vitro splatter, droplets, and aerosols in a laboratory setting with and without HVS. Location 1 reflects the dentist’s upper chest and face mask, location 2 reflects the dental assistants’ forearms and body, location 3 represents the upper portion of the patient’s chest, location 4 represents the patient’s body, location 5 represents the dental chair/suction unit. The white rectangle represents the filter paper, including the angulation and measurement distance for each of the five locations

Before each procedure, pieces of filter paper measuring approximately 150 mm × 150 mm (retention size 2.00 μm, Filtech, Wollongong, Australia) were placed in five different locations around the phantom head (Fig. 1). As shown in Fig. 1, location 1 represents the dentist's position located 20 cm away from the center of the mouth in the longitudinal plane. Location 2 was located 15 cm away from the mouth, at a 90^o^ angle to the left, to mimic a dental assistant. Location 3 was 22 cm in front of the mouth mimicking the patient’s chest, while location 4 was mimicking a location further along the patient body at 60 cm away from the mouth. Location 5 was mimicking a distant site away from the procedure 120 cm away from the center of the mouth on the left side of the patient at a 60^o^ angle. Immediately after each cycle, the filter paper was imaged for splatter, droplets, and aerosols. The filter paper locations were cleaned thoroughly at the end of each testing run, and a minimum waiting period of 30 min used between testing cycles to prevent residual effects of airborne contamination.

#### Visualization of in vitro simulated splatter, droplets, and aerosols

The splatter pattern was visualized as described previously [5]. In brief, filter paper sheets were scanned using a fluorescence imaging system (ChemiDoc MP Imaging System, Bio-Rad Laboratories Inc., USA) using the fluorescein blot filter, with a wavelength of 488 nm. Aerosol patterns at location 5 (1.2 m from the source) were imaged using an inverted fluorescent microscope at 5× magnification with a fluorescein isothiocyanate filter (model DMi8, Leica Microsystems, Japan). To obtain a true representation of the aerosol pattern at location 5, 10 random locations on the filter paper were selected for analysis for each cycle.

#### Image analysis

Images were analyzed using Fiji-ImageJ software (NIH, Wisconsin, USA) to determine the diameter of the tracer particles. The fluorescence values were calculated by converting the size-calibrated images for each device to 8-bit greyscale images, with greyscale values ranging from 0 (black) to 255 (white). The parameters measured included the percentage of total area, particle counts, median size, and Feret’s diameter. Particle spread was quantified using the percentage of the total area for each location within each AGP.

### Clinical bioaerosol spread for 19 patients following supragingival scaling

#### Ethical approval

This cross-sectional study was carried out with human ethics approval from the Metro North Hospital and Health Service (65509) and the University of Queensland (2020/HE002629). The study was conducted according to the principles outlined in the Declaration of Helsinki on experimentation involving human subjects.

#### Informed consent

Subjects were provided with detailed written and verbal information about the study and provided written consent for enrolment into the study.

#### Participant recruitment

A total of 19 patients attending the postgraduate specialist periodontal clinic between May 2021 and August 2021 for non-surgical periodontal treatment were invited to participate in the study, with no specific requirement for periodontal health status. Written consent was obtained from the participants with the following inclusion criteria: (1) ≥ 18 years old; (2) able to provide consent for enrolment in the study; (3) self-reported stable general systemic health; (4) ≥ 20 teeth (excluding third molars); (5) patients requiring supragingival debridement with an ultrasonic scaler. Exclusion criteria were (1) immunosuppression; (2) antibiotic therapy within the proceeding three months; (3) uncontrolled medical conditions; and (4) long-term use of anti-inflammatory medications. The periodontal condition of all patients was classified according to the 2017 World Workshop on the Classification of Periodontal and Peri-Implant Diseases and Conditions [20, 21].

#### Clinical procedure and bioaerosol collection

To prevent potential bias, the present study was performed by one periodontist trainee (A.P.) in three dental operatories each measuring approximately 15 m^2^ with 7 air changes per hour at the Oral Health Center Clinic, Herston, Queensland, Australia. Each room had delivery air outlets and return air collection on the ceiling. Prior to each patient appointment, all hard surfaces on the dental chair and throughout the operatory were cleaned as part of standard infection control procedures (Sani-Cloth Detergent Wipes, PDI, UK). Approximately 1 mL of unstimulated whole saliva was collected at the beginning of the appointment by asking the patients to expectorate pooled resting saliva into a sterile Falcon tube following our previously published protocols [22–25]. Following the Australian Dental Association guidelines for the COVID-19 pandemic, pre-procedure mouth rinse and high-volume suction were applied to all visiting dental patients. Fifteen milliliters of hydrogen peroxide 1.5% w/v (Colgate Peroxyl) was used for each patient to rinse for 30 s prior to the ultrasonic scaling. Aerosols, droplets, and splatter generated during the ultrasonic scaling were collected on pieces of filter paper (25 mm × 75 mm, retention size 2.00 μm, Filtech, Wollongong, Australia) that were placed at nine locations.

- Two on the patient protective sheet, either side of the midline in the upper chest area.
- Two on the dentist, on either side of the midline in the upper chest area
- Two on the dental assistant, on either side of the midline in the upper chest area
- One on the dental bracket tray table attached to the dental chair
- One on the suction unit of the dental chair
- One on the bench, approximately 1.5 m to the right of the patient
- Negative control (NC): one filter paper was not exposed during the appointment and acted as a negative control. Thus, each patient has their own NC as a background to compare.
- Whole saliva from each patient was used as a positive control.

Each patient underwent supragingival ultrasonic scaling for 10 min using the piezoelectric scaler (Piezon, KaVo, Biberach, Germany) built into the dental unit (model E-50, KaVo, Germany). The scaler was operated on a power setting of 9 with 80 mL/min of water flow rate, using a fine ultrasonic scaler tip.

After this time, the filter paper strips were collected using fresh gloves and placed into Eppendorf tubes. Within 10 min, the strips were placed in a – 80 °C freezer located in an adjacent PC2/BSL2 laboratory and then kept frozen. At the end of the clinical procedure, hard surfaces on the dental chair and throughout the operatory were again decontaminated using standard infection control procedures. There was a minimum of 60 min between patient appointments, which allowed for 7 air changes in the room. The dental team donned new protective gowns, masks, and gloves for each patient.

The protein content of each filter paper strip was determined from 25 μL eluates in 150 mmol/L phosphate-buffered saline (PBS) with a Pierce BCA Protein Assay Kit (Thermo Fisher Scientific, USA), as per the manufacturer’s instructions. Samples were incubated at 37 °C for 30 min with the test reagent, and the absorbance was measured using a microplate spectrophotometer (Infinite 200Pro, Tecan, Switzerland) at a wavelength of 560 nm. The protein quantity was normalized with a bovine serum albumin (BSA) standard. There were two aspects of data analysis: (a) bioaerosol contamination at each location for each patient was estimated based upon the protein quantity at that location and the protein concentration of the patients’ original whole saliva sample; (b) the values higher than their NC background were considered to represent contamination.

### Statistical analysis

Statistical analysis was performed using GraphPad Prism software (v9.0.0, GraphPad, San Diego, CA, USA). The Mann-Whitney *T*-test was used to assess differences between HVS and non-suction groups, with *p* values < 0.05 considered statistically significant. Particle data are presented as mean, median, and absolute counts, and error bars show standard deviations unless stated otherwise.

## Results

### In vitro simulated splatter generation with and without HVS

**Fig. 2:**
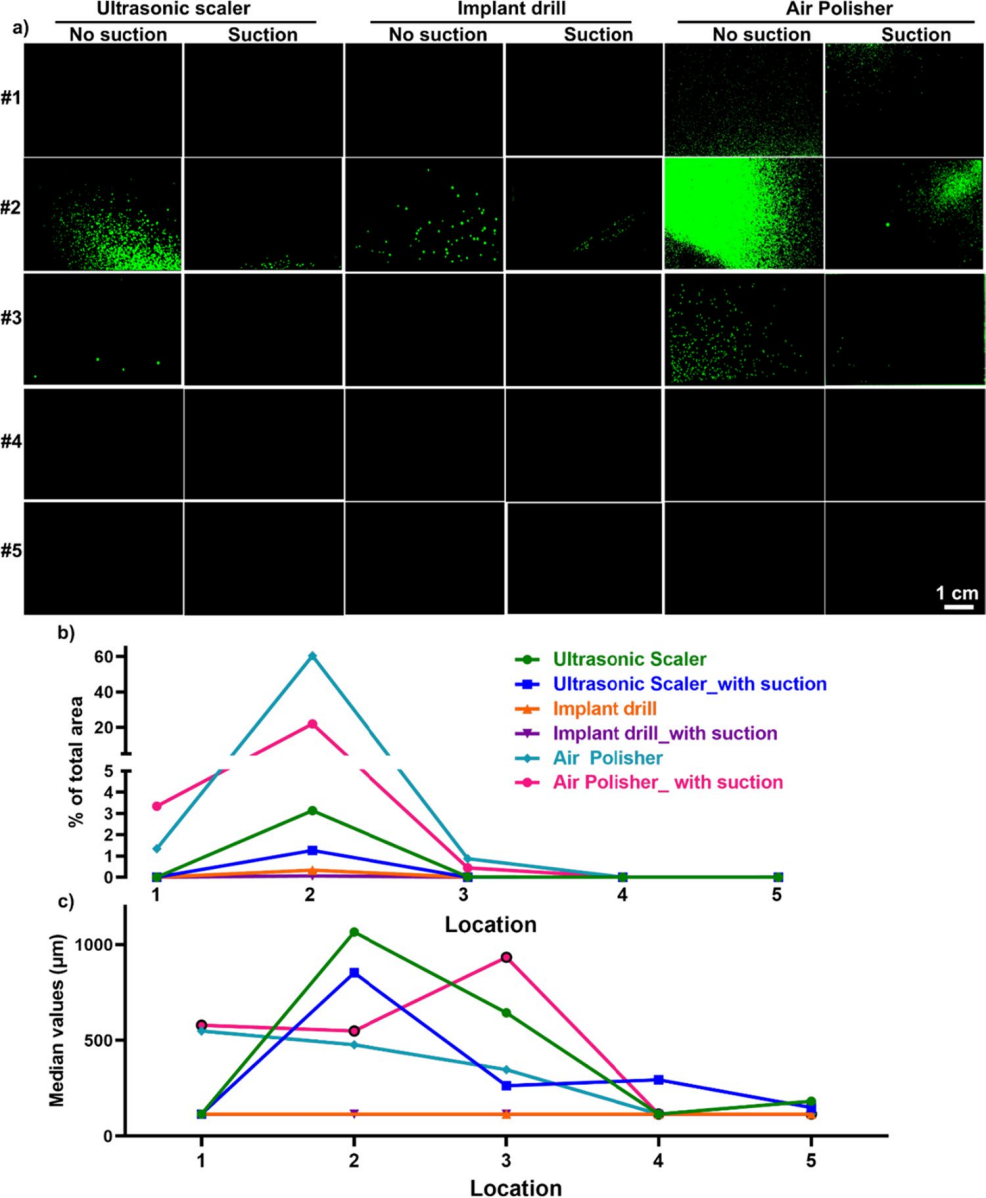
Representative images of particles on filter paper at various locations (**a**), the % of the total area (**b**), and the median particle size (**c**) for splatter particles. **a** HVS reduced splatter spread at 5 different locations: location #1 represents a right-handed dentist at 20 cm away from the source, location #2 represents a dental assistant at 15 cm away from the source, locations #3 and #4 represent the patient’s chest and body at 22 cm and 60 cm away from the source, and location #5 is at 120 cm away from the source. For locations, three dental AGPs generated the most splatter particles for a dental assistant (location 2). Among different AGPs, the air polisher generated the most splatter particles compared to the ultrasonic scaler and implant drill. **b** Quantification of splatters by dental AGPs by quantifying particles % of the total area for each filter paper. The application of HVS showed decreased splatter particles for all three dental AGPs. **c** The median size of splatter particles with and without HVS is larger than 200 μm

During 10 min of using the ultrasonic scaler on a mandibular incisor tooth, HVS reduced splatter particles for all three types of devices (Fig. 2). Locations 2 and, to a lesser extent 1 were the most spread sites for all three equipment types (Fig. 2a). It is noted that the 2-min air polisher generated more splatter particles compared to a 10-min ultrasonic scaler procedure, while the 2-min implant drill led to the least splatter liquid particles. The number of particle numbers was quantified by measuring the percentage of the total area of each filter paper. HVS reduced the extent of spread for all three dental AGPs (Fig. 2b). The distribution of particle size at the five locations exhibited median values larger than 200 μm (Fig. 2c), consistent with splatter spread (large droplets).

**Fig. 3:**
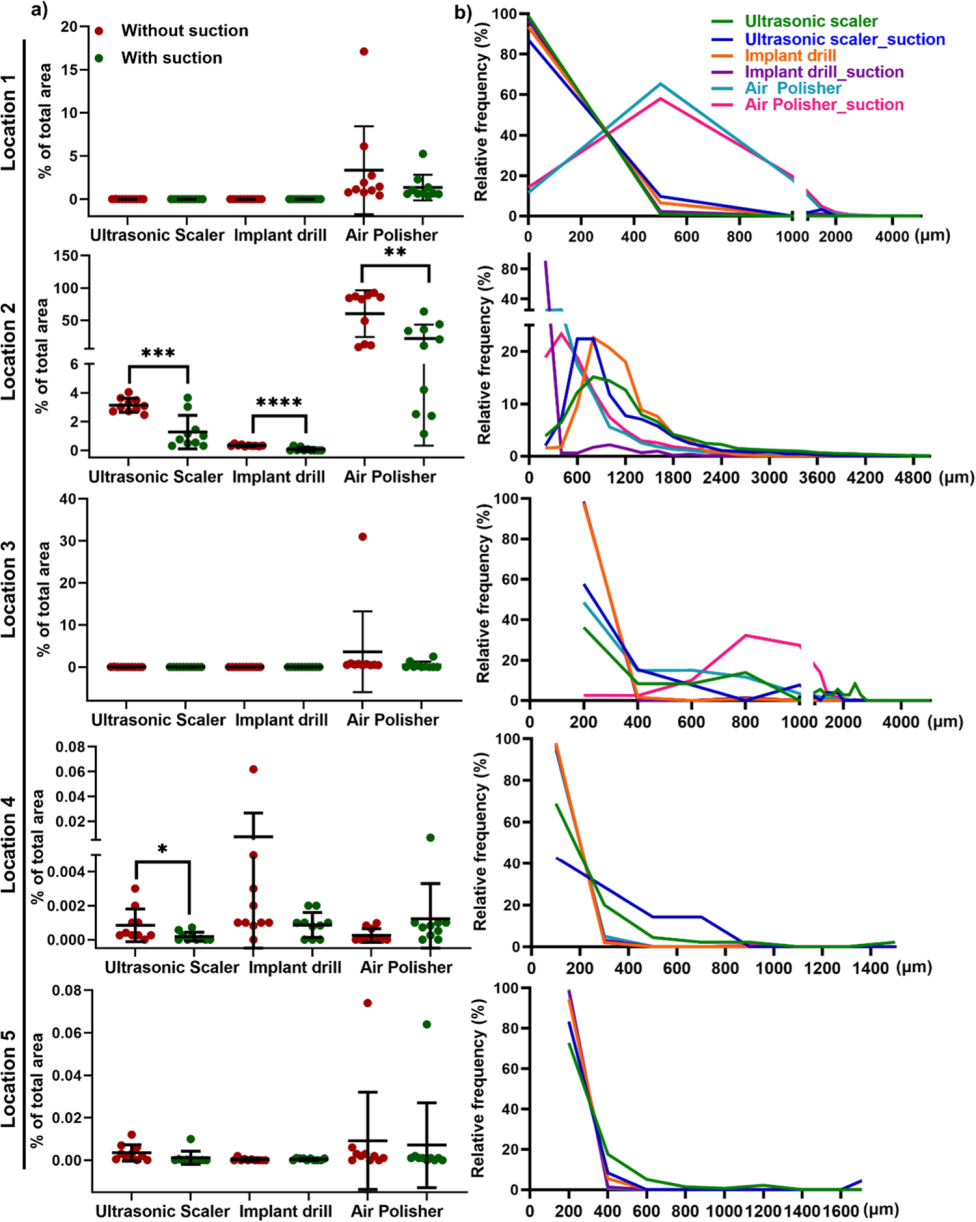
Quantification of splatter spread with/without HVS expressed as a percentage of the total area (**a**) and the histogram patterns of particle size (**b**). **a** Splatters quantification by calculating the % of the total area of each filter paper for every dental AGP with and without HVS for 5 locations. Each dot indicates an independent experiment. The HVS significantly reduced splatters for all three dental AGPs at location 2. At location 4, HVS significantly decreased splatter particles for ultrasonic scaler. Data are displayed as mean values ± SD. *p < 0.05; **p < 0.002; ***p < 0.0002; ****p < 0.0001 between with HVS and without HVS. **b** The use of HVS did not change the histogram of splatter particle size for all locations. For locations 1–3, splatter particles peaked at ~ 600 μm, while particles were smaller (peaked at 300–400 μm) at locations 4–5. This indicates large particles mainly deposit at the dentist, dental assistant, and patient’s chest

Particle numbers (Fig. 3a) and distributions (Fig. 3b) were measured for each location. A significant benefit for the use of HVS was seen with all devices at location 2, as well as for the ultrasonic scaler at location 4 (Fig. 3a). Particle histogram patterns at all five locations demonstrated that HVS did not alter the median size of splatter particles (Fig. 3b). A significant reduction in splatter spread for the ultrasonic scaler was observed with HVS when all particles for all five locations were combined (Supplementary Table 1). It is noted that up to 90% of splatter reduction was observed with HVS at location 2 for the air polisher (Supplementary Table 1).

### Aerosol and droplet particles at 120 cm away from the source with and without HVS – in vitro

**Fig. 4:**
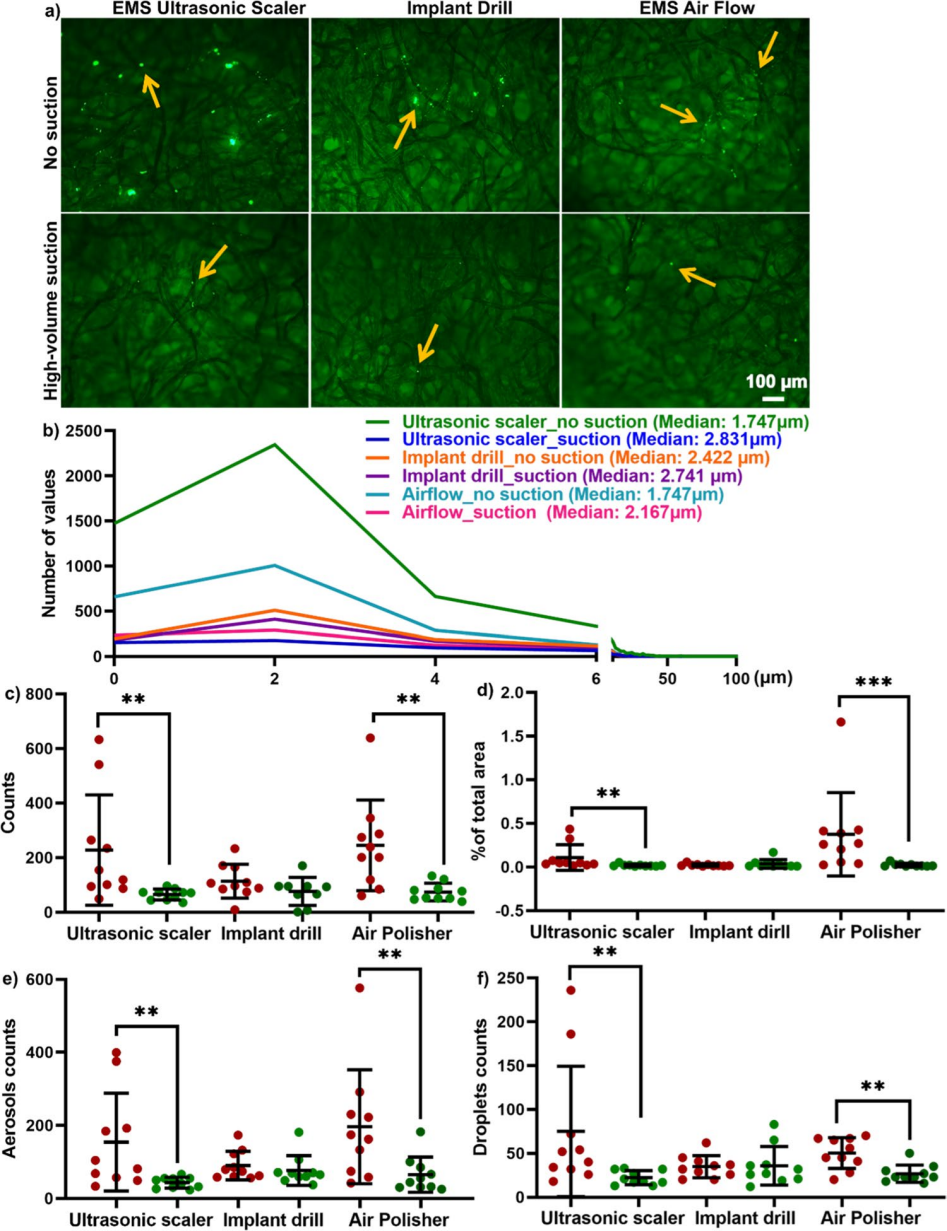
Representative images (**a**), histogram (**b**), and quantifications (**c**–**f**) of aerosol and droplet spread at 1.2 m away from the source. **a** The application of HVS significantly reduced the aerosol and droplet particles that were retained on the filter paper fiber at 1.2 m away from the source. Yellow arrows indicate fluorescein-stained particles on filter paper fibers. **b** The histogram of aerosol and droplets particles (ranging from 0.7 to 100 μm) was detected at 1.2 m away from the source. The median size of aerosol/droplets was smaller than 3 μm and the use of HVS reduced aerosol/droplet particles for all three dental AGPs. **c**–**d** HVS significantly reduced the number of mixed aerosols/droplets by counts per image (**c**) or as a percentage of the total area (**d**) of each filter paper. **e**, **f** When the data were separated into aerosols (**e**; ≤ 5 μm) and droplets (**f**; > 5 μm), the HVS significantly decreased the aerosol (**e**), and droplets (**f**) particles for ultrasonic scaler and air polisher. Data in **c**–**f** are displayed as mean values ± SD. **p < 0.0002; ***p < 0.0002 between with and without HVS

The aerosols and droplets that were retained on filter paper fibers were captured at 120 cm from the source (location 5) after 10 min of the ultrasonic scaler and 2 min of air polisher and implant drill (Fig. 4a). Small particles (0.7 to 100 μm in diameter) were detected, whether or not HVS was used, indicating a mixture of aerosols and droplets (Fig. 4b). The ultrasonic scaler produced the highest number of particles that were 5 μm in median diameter or less. The use of HVS reduced particle quantity for all three devices.

Analysis of the average particle count (Fig. 4c) and the percentage coverage of the total area (Fig. 4d) for a mixture of aerosol and droplets revealed that HVS significantly reduced 82.6% and 93.8% of small particles at location 5 for both the ultrasonic scaler and the air polisher ( Fig. 4c, d). The same was found for a separate analysis of aerosol (Fig. 4e) and droplet particles (Fig. 4f).

Taken together, the in vitro simulated studies demonstrated that the air polisher generated most splatter particles and the use of HVS significantly reduced the spread of splatter, droplets, and aerosols for ultrasonic scaler and air polisher.

### Bioaerosol contamination in a clinical setting during routine periodontal supragingival scaling

Whether bioaerosol spread can generate hazards to dental health professionals in a clinical setting was examined. A total of 19 patients (1 healthy – BOP < 10%; 3 gingivitis - BOP > 10 % and 15 periodontitis – 2 × stage 1, 10 × stage 3, 3× stage 4) requiring supragingival calculus removal as part of their dental care were recruited, thus generating a total of 190 clinical (filter paper) samples and 19 saliva samples. The patient characteristics are shown in Table 1. The clinical study included 9 females and 10 males, aged 63.3 ± 13.2 years old (ranging from 35 to 80 years old) with one smoker. The average PPD for all patients ranged from 2.34 to 3.27 mm, with an average of BOP of 18% ± 12.2% (ranging from 4 to 44%) and PI of 22.9% ± 11.3 % (ranging from 2 to 42%). For periodontitis patients, 2.42 ± 3.06 sites had a deep periodontal pocket that is ≥ 5 mm (ranging from 0 to 13).

**Table 1:** Patient demographics and periodontal status

Patient	Gender	Age	Periodontal health status (classification)	Smoker status	Average PPD (mm)	Number of sites ≥ 5 mm	BOP	PI
1	Male	80	Stage 1 grade B	No	2.57	1	44%	31%
2	Female	65	Stage 3 grade B	No	2.84	5	14%	20%
3	Female	67	Stage 4 grade C	Yes	2.52	4	28%	41%
4	Female	68	Stage 3 grade B	No	2.81	2	15%	26%
5	Female	72	Stage 1 grade B	No	2.35	0	11%	6%
6	Male	41	Stage 3 grade B	No	2.90	5	26%	42%
7	Male	73	Gingivitis	No	2.74	0	27%	38%
8	Female	35	Ginivitis	No	2.49	0	25%	21%
9	Female	72	Stage 4 grade B	No	2.62	2	5%	23%
10	Male	74	Stage 3 grade B	No	2.47	1	13%	26%
11	Female	74	Stage 3 grade B	No	2.88	3	12%	18%
12	Female	42	Gingivitis	No	2.55	0	20%	17%
13	Male	65	Stage 4 grade B	No	2.68	0	6%	2%
14	Male	60	Stage 3 grade C	No	2.47	2	4%	14%
15	Male	67	Stage 3 grade B	No	2.88	3	31%	34%
16	Male	69	Healthy	No	2.29	0	3%	18%
17	Male	50	Stage 3 grade B	No	2.34	2	10%	8%
18	Female	51	Stage 3 grade B	No	2.58	3	40%	30%
19	Male	77	Stage 3 grade B	No	3.27	13	8%	20%

Abbreviations: *PPD*, periodontal probing depth; *BOP*, bleeding on probe; *PI*: plaque index

**Fig. 5:**
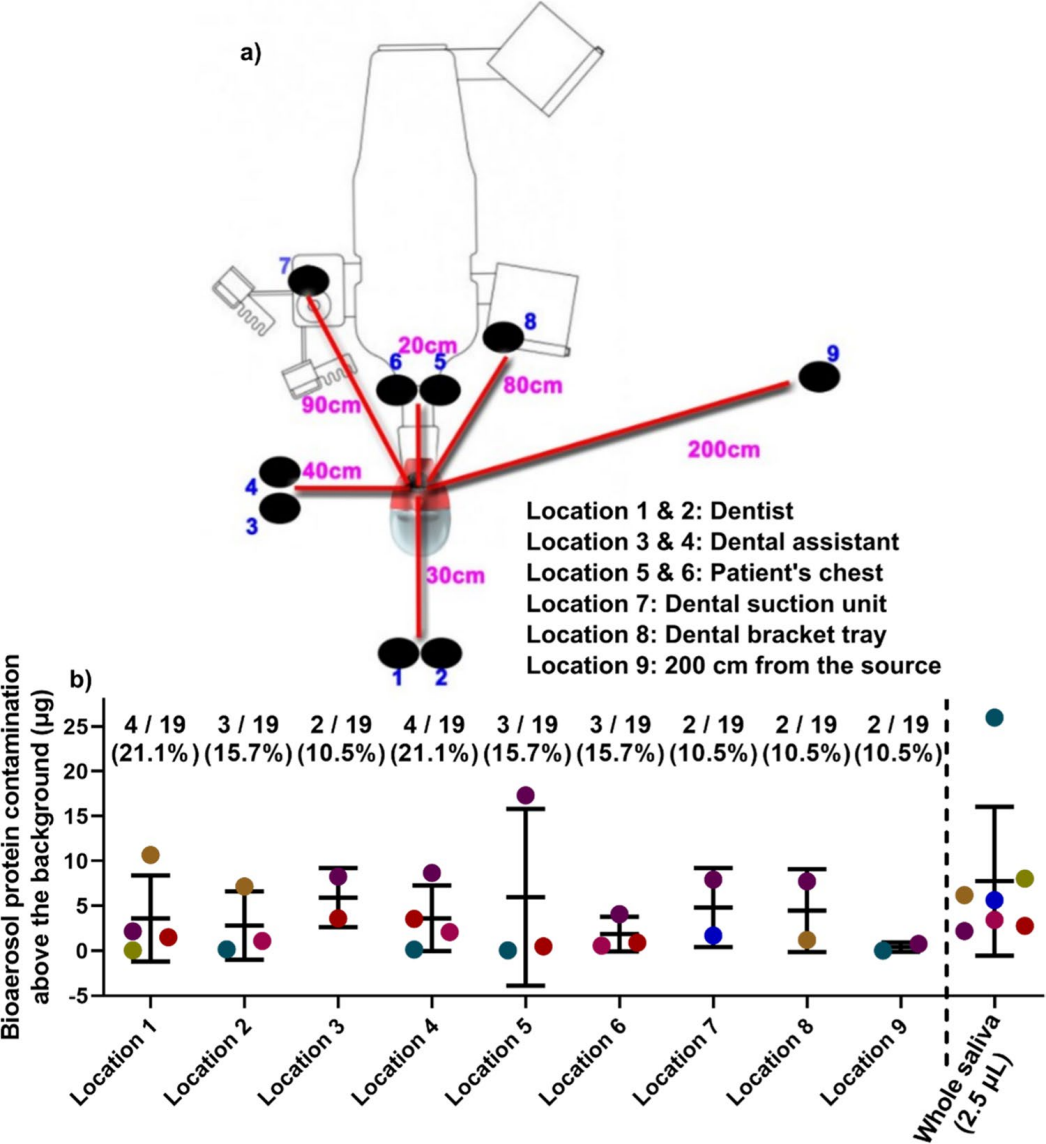
Clinical bioaerosol collection from 9 different locations (**a**) and only 10.5–21.1% of patients showed positive bioaerosol protein contamination (**b**). **a** Locations 1 and 2 represent the dentist located at 30 cm away from the patient’s mouth; locations 3 and 4 represent the dental assistant at 40 cm away from the patient’s left side; locations 5 and 6 represent the patient’s chest at 20 cm away from patient’s mouth; location 7 represents the dental suction unit at 90 cm away from the patient’s mouth; location 8 represents the dental bracket tray at 80 cm away at the patient’s left side; and location 9 represents a storage area on the side of the room located 200 cm away from the patient’s mouth on the patient’s right side. **b** Dot plot graph showed positive bioaerosol protein contamination for those who showed positive bioaerosol contamination that was above the background at various locations. Only 10.5% - 21.1% of patients showed positive bioaerosol contamination at any particular site. For instance, compared to the background negative controls, 4 out of 19 patients at location 1 produced bioaerosol protein contamination. The protein content of 2.5 μL of whole saliva from each patient (right of dotted line) was included as a reference, demonstrating that there was significant protein content in even small amount of saliva (2.5 µL), albeit this was variable between different patients. Each coloured dot point indicates an individual. Y aixs values = the protein quantity at each location from each patient – background negative control. Data are displayed as mean ± SD

Samples were eluted from filter paper strips placed at 9 different locations (Fig. 5a). Compared to each patient’s background (NC filter paper), protein quantification at each location showed that only 10.5–21.1% of patients generated bioaerosol protein contamination beyond the relevant negative control sample for each patient (Fig. 5b and Supplementary Fig. 1a). The extent of protein contamination at each location varied between patients and was not influenced by periodontal health status (Supplementary Figure 1b). Under the same routine supragingival scaling for 10 min, it was noted that patients #1, #5, #6, #7, #10, # 14, and #18 had protein contamination above background levels at some locations (Fig. 5b). After this pilot study, all patients underwent routine non-surgical periodontal treatment (for periodontitis patients).

## Discussion

This study showed the particle distribution following three diverse dental AGPs, with airflow creating the most particles, while the implant twist drill produced minimal particles across all three categories (splatter, droplets, and aerosols). The use of HVS could effectively reduce up to 98% of splatter, aerosol, and droplets particles in a simulation study. In a clinical setting, our data suggest that protein contamination varied between individuals, with less than 20% of patients generating bioaerosol protein contamination during routine supragingival scaling treatment.

It is worth highlighting that the present study adds new information to the “dental aerosol spread” field: (a) we utilized a simple fluorescence tracer to visualize how in vitro stimulated aerosol and droplets were reduced by HVS during dental AGPs, especially for air polisher and low-speed implant drill (less studied in the field); (b) our clinical study investigated bioaerosol distribution patterns following supragingival scaling procedures via a simple protein test, which is distinct from most current studies that use microbial presence as an indicator of clinical bioaerosol contamination. Our study provided alternative ways to explore dental AGPs generated aerosol and droplets travel in vitro and clinically, which facilitates future dental research. Our in vitro simulated study investigated splatter, droplets, and aerosol spread with and without HVS for common dental AGPs—ultrasonic scaler, implant drill, and less investigated air polisher.

The results of the present study demonstrated that air polishing even after a 2-min procedure, with or without HVS, was the highest producer of splatter particles (Table 1), with higher splatter particle generation than a 10-min ultrasonic scaler procedure, which is consistent with previous reports [8, 26]. Furthermore, our data confirmed that aerosol and droplet particles can travel up to 1.2 m from the source (Figs. 3 and 4), with ultrasonic scaler generating the most aerosol and droplet particles. The present results showed that median particles less than 5 μm in size can be found 1.2 m from the source, even for a procedure such as an implant osteotomy preparation where there is no pressurized air involved. Such results are in agreement with previous studies [26–29]. This is similar to the conclusions of Graziani et al., who found that ultrasonic scaling without suction produced particles ranging from 0.3 to 10 μm in size, measured at 1 m from the source [29]. Kaufmann et al. demonstrated the particles were detectable up to 1.1 m and 1.2 m away when using an ultrasonic scaler and an air-polishing unit, respectively [26]. Moreover, Legnani et al. showed that a procedure that combines an ultrasonic scaler with air polishing produced particles traveling up to 1.5 m away from the source [27, 28]. This is perhaps not surprising considering the mix of pressurized air, water, and non-abrasive powders that are designed to travel at great velocity onto the tooth, whereas for ultrasonic scalers the air and water are designed to work together at the instrument tip.

The influence of HVS on particles generated by dental procedures is typically measured using culture-based methods focusing on aerobic bacteria, rather than on salivary proteins, as in the present study. The correct use of HVS has been shown to reduce the level of bacteria in bioaerosols during and after the procedure [28, 30, 31]. The present results showed that 37.5–96% of splatters were reduced by HVS for three dental AGPs at various locations. It is worth noting that up to 96% of splatters by ultrasonic scaler were reduced by HVS at location 1 (dentist). This confirmed the importance of using HVS for standard dental procedures that generate splatter particles, for the three different devices which were used. This finding is in line with the results of previous studies [13, 32]. King et al. showed that HVS causes a 50 to 90% reduction in bacterial levels when used in conjunction with ultrasonic scaling [32]. Holliday et al. reported a reduction of 60% [13], which is identical to the finding in the current study for ultrasonic scaling at location 2. However, present studies either use medium-volume suction [13] or HVS with an unstated speed [32]. Instead, the present study used an HVS at the speed of 325 L/min and this is a real HVS according to current international guidelines. Furthermore, HVS reduced 82.6% and 93.8% of droplets and aerosol particles for ultrasonic scaler and air polisher, respectively, which supports the previous study that aerosols (≤ 5 μm particles) were reduced by HVS [17]. These findings support the view that using HVS during dental AGPs can dramatically reduce the spread of particles and thereby reduce the risk of occupational infection among dental healthcare workers.

Our clinical study indicated that there are less than 20% of patients generated bioaerosol protein contamination in the particles generated by ultrasonic scalers (Fig. 5). However, when looking at individual patient data, there was individual variation between the sampling locations and negative control. During 10 min of ultrasonic scaling, saliva might not be the main source of protein contamination, and this becomes mixed with the coolant water spray [11]. This is consistent with the findings of Meethil et al., who showed that fluids recovered at large distances from the oral cavity are comprised of coolant water rather than material from the patient’s mouth and that saliva contributes very little to the microbes present in aerosols generated during ultrasonic scaling [10]. It is worth noting that patients in the present study were treated in a normal manner with an HVS and a pre-procedure mouth rinse was applied for each patient as per dental guidelines. This may explain mouth rinse and HVS reduced protein content that was explained by previous studies using bacterial CFUs [18, 33]. Additionally, we noticed a high level of background protein contamination for the filter paper negative controls. Since the filter paper was handled with gloves, the source of the trace elements is unclear but it may have occurred during the manufacturing process, subsequent handling, the atmosphere, or a combination of these. Nevertheless, it is noted that only less than 4 patients (out of 19 patients) showed bioaerosol protein contamination with a higher protein quantity above negative control (filter papers). It is well accepted that a healthy patient’s saliva flow rate is 0.3–0.4 mL per min [34]. Our data indicate that non-surgical periodontal treatment procedure - supragingival scaling for 10 min might not pose a significant danger for dental professionals for transmission of SARS-CoV-2 virus. Further work is needed to determine if the protein content recovered on filter paper strips is sufficiently sensitive to correlate precisely with the presence of viruses or bacteria of salivary origin. However, it is challenging to detect virome using a universal primer and obtain sufficient virus load from the oral cavity. Employing PCR or sequencing can be informative in terms of identifying the most likely source for microbial contaminants that are found in the dental surgery environment since these may have come from the environment, equipment waterlines, or patients. Finally, the present study has several limitations that could impact the interpretation of the results. The in vitro study model could be extended by having more sampling locations around the typodont to assess the spatial distribution of the fluid movement study. The locations for procedures were deliberately chosen to be on anterior teeth. Procedures performed on posterior teeth would likely show different patterns of splatter, droplets, and aerosols, according to the orientation of the device used in the AGP. Secondly, the clinical study did not measure the level of specific microorganisms recovered on the filter paper. Thirdly, our in vivo bioaerosol study did not compare bioaerosol distributions between implant drill and airflow polisher. Further studies comparing different common periodontal treatment procedures and utilizing 16s sequencing would be useful to confirm the presence of saliva-derived microorganisms and track the level of these versus the protein content. Another limitation is that the in vitro simulated study used a closed room (with air-conditioning outlet and air-conditioning intake located in the same room), which is not the normal situation for a dental practice as often the return air (air intake) is located outside in the corridor.

## Conclusion

The in vitro simulated component of the study shows that, within the limits of the model used, the air polisher produced the largest amount of splatter particles, while the ultrasonic scaler generated the largest amount of aerosol and droplet particles at 1.2 m away from the source. The use of HVS can reduce up to 96 % of splatters and 93% of aerosol and droplets spread. Moreover, a 10-min period of supragingival ultrasonic scaling in less than 20% of patients produces protein contamination that is above the background, suggesting that supragingival ultrasonic scaling may not produce significant amounts of bioaerosol contamination in the majority of clinical cases.

## Supplementary Information

ESM 1 (DOCX 231 kb)
