# Supplementary material for: Simulated and clinical aerosol spread in common periodontal aerosol-generating procedures
Source: Clin Oral Investig. 2022 May 17;26(9):5751–62. doi: 10.1007/s00784-022-04532-8 (PMC9113070; doi:10.1007/s00784-022-04532-8)
Supplement: Supplementary file 1 — (DOCX 231 kb) [file 784_2022_4532_MOESM1_ESM.docx]

**Supplementary materials for**

**Simulated and Clinical Evaluation of Particle Spread Associated with Common Periodontal Aerosol-Generating Procedures**

**Supplementary Table 1. Percentage coverage of the total area by particles for each location and the sum of all tested locations for each AGP (mean±SD)**

|  | AGP | | | | | |
| --- | --- | --- | --- | --- | --- | --- |
| Location | U/S | U/S w S | A/P | A/P w S | Imp | Imp w S |
| 1 | 0.0171 ±  0.0038 | 0.0006 ±  0.0008  # 96.5 % | 3.3448 ±  4.8459 | 1.3442 ±  1.4046  # 59.5 % | 0.0010 ±  0.0014 | 0.0006 ±  0.0006  # 40 % |
| 2 | 3.1372 ±  0.4621* | 1.2697 ±  1.1032  # 59.5 % | 60.3766 ±  34.1727* | 21.9574 ±  20.5206  # 63.6 % | 0.3373 ±  0.0616* | 0.0673 ±  0.1174  # 80.1 % |
| 3 | 0.0208 ±  0.0328 | 0.0041 ±  0.0101  # 80.3 % | 3.6207 ±  9.1201 | 0.4502 ±  0.7960  # 87.6 % | 0.0008 ±  0.0006 | 0.0005 ±  0.0005  # 37.5 % |
| 4 | 0.0008 ±  0.0009* | 0.0002 ±  0.0002  # 75 % | 0.0077 ±  0.0182 | 0.0009 ±  0.0007  # 88.3 % | 0.0002 ±  0.0004 | 0.0012 ±  0.0020 |
| 5 | 0.0035 ±  0.0036 | 0.0011 ±  0.003  # 68.5 % | 0.0003 ±  0.0006 | 0.0004 ±  0.0004 | 0.0091 ±  0.0217 | 0.0071 ±  0.0190 |

U/S = ultrasonic scaler; A/P = air polisher; Imp = implant osteotomy. Adjacent columns show the AGPs without suction and then with suction. # mean reduction % after HVS. *: significant difference without and with HVS.


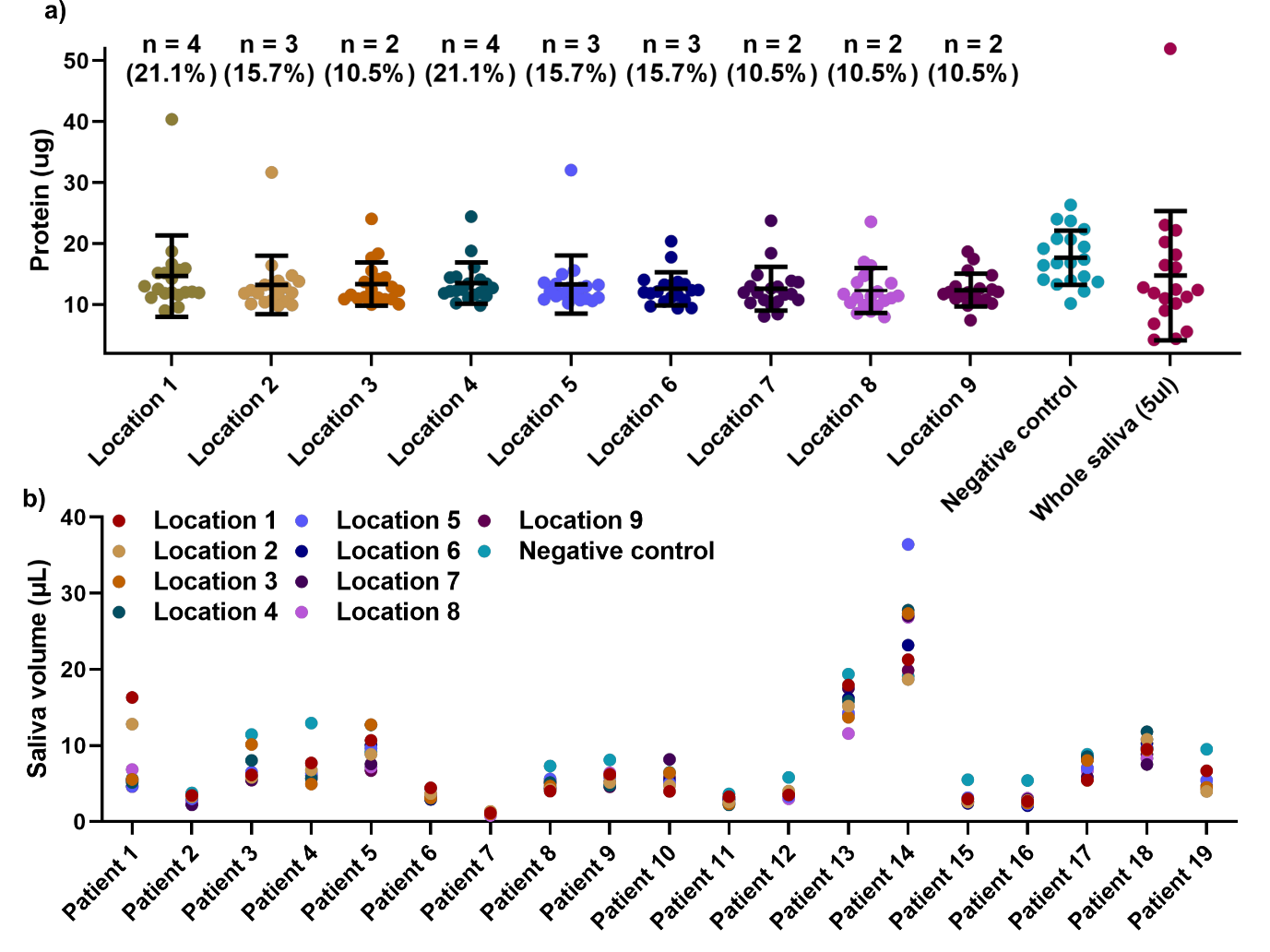


**Supplementary Figure 1. Bioaerosol protein content for each patient.** a) Each dot represents data from individual patients. The protein content of the negative control and 5 µL of whole saliva is included as a reference point. Data are displayed as mean ± SD. The percentage value on top of each location reflects the proportion of patients that produced detectable protein contamination. b) The saliva contamination volume at each location for each patient was calculated using the protein quantity at the location and the known protein concentration of the whole saliva sample from that same patient.
